# Synergic effects of *Trigonella foenum-graecum, Ribes rubrum, Lavandula angustifolia*, and *Arctium Lappa* extracts mixture on learning and memory deficits in streptozocin-induced diabetic rats

**DOI:** 10.22038/IJBMS.2023.70096.15246

**Published:** 2023

**Authors:** Mona Sadat Hosseini Arya, Samaneh Kakhki, Farimah Beheshti, Mohammad Hadi Ebrahimzadeh, Jalil Farzadmehr

**Affiliations:** 1 Student Research Committee, Torbat Heydariyeh University of Medical Sciences, Torbat Heydariyeh, Iran; 2 Department of Clinical Biochemistry, School of Medicine, Torbat Heydariyeh University of Medical Sciences, Torbat Heydariyeh, Iran; 3 Neuroscience Research Center, Torbat Heydariyeh University of Medical Sciences, Torbat Heydariyeh, Iran; 4 Departments of Physiology, School of Medicine, Torbat Heydariyeh University of Medical Sciences, Torbat Heydariyeh, Iran; 5 Department of Plant Production, Faculty of Agriculture, University of Torbat Heydarieh, Torbat Heydarieh, Iran; 6 Faculty of Agriculture, University of Torbat Heydarieh, Iran

**Keywords:** Arctium lappa, Diabetes mellitus Lavandula, angustifolia Ribes, khorasanicum, Streptozotocin, Trigonella foenum-graecum

## Abstract

**Objective(s):**

The present study aimed to assess the efficacy of some known extracts on learning and memory impairment induced by streptozocin (STZ) in male rats.

**Materials and Methods:**

Eighty male rats were randomly divided: 1) control, 2) STZ (50 mg/kg), 3) STZ+*Trigonella foenum-graecum* (200 mg/kg), 4) STZ+*Ribes rubrum* (500 mg/kg), 5) STZ+*Lavandula angustifolia* (400 mg/kg), 6) STZ+*Arctium Lappa* (200 mg/kg), 7) STZ+mix of extracts (quarter dose of each extract), and 8) STZ+metformin (100 mg/kg). Treatment was continued for 8 weeks and the after that, the behavioral tests related to learning and memory including Morris water maze (MWM) and passive avoidance (PA) were performed along with biochemical analysis associated with oxidative stress pathway and other related indicators.

**Results:**

According to the results, all extracts demonstrated potential effect to ameliorate cognitive impairment caused by STZ in both MWM and PA tests along with attenuating oxidative stress indicators like malondialdehyde (MDA), while total thiol content and anti-oxidant enzyme activity like superoxide dismutase (SOD) and Catalase (CAT) remarkably increased in biochemical test results. Interestingly, the mixture of extracts illustrated much better results in ameliorating the brain-derived neurotrophic factor (BDNF), while attenuating the amyloid-B and glial fibrillary acidic protein (GFAP).

**Conclusion:**

The present study demonstrated these extracts alone or in combination with a minimum dose have a strong potential to ameliorate learning and memory impairment induced by STZ along with lowering glucose levels by which they prevent or manage diabetes. It is noteworthy that the results matched those of metformin a well-known anti-diabetic drug.

## Introduction

Diabetes mellitus (DM) as a prevalent chronic metabolic disorder is categorized into three main groups type 1, type 2, and gestational diabetes mellitus (GDM) which is mainly related to lack of insulin secretion, decreased cell response to insulin which results in elevated level of serum glucose (1-3). It is considered a syndrome that involves many organs leading to numerous disorders including vascular function disorder, neuropathy, central nervous impairment, and key organ damage such as kidney, heart, and brain which is associated with health care costs (4, 5). In fact, numerous lines of literature demonstrated the higher probability of dementia and cognitive dysfunction in individuals with DM (6-8). The leading cause of cognitive disorder is more likely vascular atherosclerosis induced by insulin and blood sugar dysregulation (9, 10). Further, insulin has a pivotal neuroprotective role in the brain through apoptosis and oxidative stress, and hyperglycemia is also considered a toxic mediator on neurons through overproduction of oxidative stress indicators like MDA or pro-inflammatory cytokine (11-14); although the exact mechanism is unknown and needs more investigation. According to the global diabetes prevalence in 2019, it is estimated around 9.3% (463 million people) are suffering from DM worldwide, and constantly increasing which is expected to reach 10.2% (578 million) and 10.9% (700 million) by 2030, and 2045 (15), respectively. In addition, the total cost of diabetes was estimated at 2.4 billion dollars in 2010. In fact, according to IDF Diabetes Atlas, global diabetes-related health expenditures were estimated at around 966 billion USD in 2021, and are expected to reach 1045 billion USD by 2024 (16, 17). 

In order to reduce the risk of these complications, the early detection and management of diabetes and pre-diabetes is imperative. There is strong evidence demonstrating that DM significantly decreased learning and memory which affects animal and human capacity to have difficulties in learning and remembering through different mechanisms which are mainly via overproduction of oxidative stress in the hippocampus or increasing apoptosis leading to significant reduction in neurons (18, 19). As would be expected, early diagnosis and appropriate treatment significantly reduced both healthcare costs and complications associated with diabetes, so, discovering and presenting new medicine with the least adverse effects needs more attention (20, 21). 

There are a variety of oral and parenteral medicines which increase insulin secretion or cell response, however, due to therapy failure, numerous attempts have been undertaken in recent years to find alternatives especially among herbal remedies, and plant extracts such as *Lavandula angustifolia* (Lavandula), and *Trigonella foenum-graecum* (Trigonella) (22-24). In this regard, some studies have shown the anti-diabetic effects of Lavender and Trigonella; however, to the best of the authors’ knowledge, the anti-diabetic effect of two other extracts have been studied less, therefore, assessing the efficacy of them alone or mixture should be considered (25, 26). Two other extracts whose anti-diabetic effects have been less studied are *Ribes rubrum* (Ribes) and *Arctium Lappa* (Arctium). These extracts have also shown that they can have beneficial effects on diabetes (27, 28).

Considering the rich and diverse source of herbal medicine in Iran, the present study aimed to evaluate the effect of *L. angustifolia*, *A. lappa*, *T. foenum-graecum*, and *Ribes khorasanicum* and their combination with lower dose administrated alone on serum blood glucose and memory impairment induced by streptozotocin (STZ).

## Materials and Methods

Eighty healthy Wistar rats (210-250 g) were provided by the animal house of Torbat Heydariyeh University of Medical Sciences under standard laboratory settings (average room temperature of 23±2 ^°^C, 12 hr dark/12 hr light cycle) with unlimited access to food and water, and randomly divided into eight groups (n=10/group) which were classified into three main groups as follows:

1. Control: Rats received (1 ml/kg) normal saline intraperitoneally.

2. Diabetic rats: Diabetes was induced by STZ (50 mg/kg) intraperitoneally.

3. Diabetic rats-Lavandula: Diabetic rats received an extract of *L. angustifolia* (400 mg/kg) intraperitoneally (29).

4. Diabetic rats-Trigonella: Diabetic rats received an extract of *T. foenum* (200 mg/kg) intraperitoneally (30). 

5. Diabetic rats-Arctium: Diabetic rats received an extract of *A. lappa* (200 mg/kg)

intraperitoneally (31).

6. Diabetic rats-Ribes: Diabetic rats received an extract of *R. khorasanicum* (500 mg/kg) intraperitoneally (27). 

7. Diabetic rats-mixed: Diabetic rats received (a quarter of the dose administrated alone) a combination of all extracts intraperitoneally.

8. Diabetic rats-metformin: Diabetic rats received metformin (100 mg/kg) intraperitoneally. 

Treatment was continued for four consecutive weeks intraperitoneally. To induce the diabetic model, the desired animals were deprived of food for 12 hr before STZ injection, and 2 hr after injection, they returned to a normal diet as before. At the end of the study, blood samples were taken from the tail of each rat to analyze blood plasma glucose, and then animals were prepared for behavioral testing including Morris Water Maze (MWM) and Passive Avoidance (PA), eventually they were anesthetized and sacrificed to remove cortex and hippocampus tissue to measure biochemical parameters.

***Extracts and preparation***

The flower of *L. angustifolia*, root of *A. lappa*, fruit of *R.* *khorasanicum*, and the seeds of *T. foenum-graecum* were procured from a local market which were identified and confirmed by the Herbarium School of Pharmacy, Mashhad University of Medical Sciences, Iran. The mentioned parts of herbal plants were dried, milled, and turned into powder. Ethanoic extracts of Lavandula, and Trigonella with methanolic and ethyl acetate extracts of Ribes and Arctium plants weighing 250 gr were mixed and Erlenmever flasks were placed on the heater-shaker for 24 hr. The mixtures were filtered, and evaporated under reduced pressure to dryness.

***Behavioral tests***

*Morris Water Maze (MWM)*

MWM is widely used as one of the golden standards to assess spatial memory and learning especially in rodents which consists of a swimming pool (diameter is 150 cm and the height is 60 cm) with a hidden platform filled with 22-26 ^°^C water. For five consecutive days, all animals were located in different parts of the pool and allowed to swim to find the hidden platform for 90 sec. In the event, the animals could not find the hidden platform, they were gently guided toward the platform and stayed for 30 sec. Finally, on the sixth day as a probe test, to assess spatial learning and memory, the hidden platform was removed and they were allowed to find the platform for 60 sec the video camera recorded movement, time latency, and the time spent in the target quadrant (32).

*Passive avoidance (PA)*

In this experimental protocol, the animals learned to avoid an environment in which they had faced an aversive stimulus. In this method, the test apparatus comprises two light and dark compartments separated by a guillotine door. In the first step, the guillotine door was opened and animals were freely allowed to explore throughout the apparatus for 5 min. Then, an electric shock (2 mA, 2 sec) was applied to the animals’ feet as soon as they entered the dark chamber (acquisition phase). After 1/24/48 hr, the animals were transferred to the light compartment. Finally, the duration of the delay to enter the dark section (latency) alongside the duration of stay in darkness and the number of entries into the dark section were recorded for each rat (32). 

***Biochemical assessments***

*Malondialdehyde (MDA)*

The hippocampal concentration of MDA, as a marker of lipid peroxidation, was measured as described previously. MDA reacts with thiobarbituric acid (TBA) which produced a red chemical complex measured at maximum absorbance (=523 nm). 

*Total thiol (SH) contents*

Total thiol content was measured using a method in which the reaction occurs between DTNB (2, 2’-dinitro-5, 5’-dithiol benzoic acid) and thiol groups to form a yellow chemical substance. In the final step, the absorbance index was obtained at =412 nm (33). 

*Superoxide dismutase (SOD) and Catalase activity*

The Madesh and Balasubramanian process was used to measure the SOD activity at 570 nm according to a colorimetric technique (34). One unit of SOD was equal to the amount of enzyme that should be inhibited by 50% of the MTT reduction rate. The Aebi method was employed to measure CAT activity using hydrogen peroxide (30 mM) as a substrate (35).

*Blood sugar*

To measure the serum glucose level, the colorimetric method was used by which the quinonimines produced through oxidative reaction demonstrated a direct association with glucose (36).

*Assessment of Brain-Derived Neurotrophic Factor (BDNF), Glial fibrillary acidic protein (GFAP), and Amyloid beta (amyloid-B)*

Concentrations of BDNF, GFAP, and amyloid-B were measured using rat ELISA kits (IBL International, Hamburg, Germany and MyBioSource, San Diego, CA, USA), based on the manufacturer’s instructions. A microplate reader (Biotech, USA) was used and absorption rates were recorded and compared with the standard curve in the same experiment.

***Statistical analysis***

The statistical analysis of data was performed using the SPSS v.16 software package. All the results were expressed in means±standard error and an alpha (*α*) at 95% confidence interval (*P*-value=0.05) was considered for statistical significance. One-way ANOVA and Tukey’s *post hoc* tests were utilized to evaluate the behavioral and biochemical data.

## Results

***A mix of extracts improved blood sugar levels in diabetic rats***

**Figure 1:**
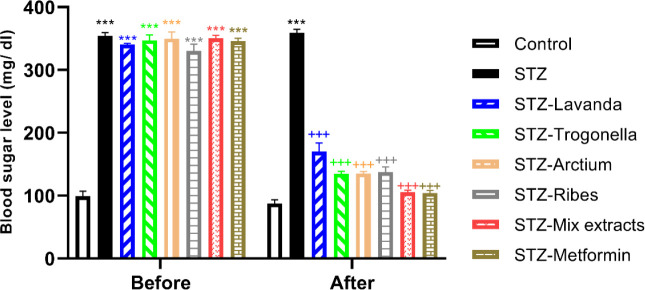
Comparison of blood sugar levels between groups

Based on the results, blood glucose in all diabetic groups significantly demonstrated higher levels than controls (*P*<0.001, for all groups). Also, the STZ group demonstrated a significant difference from the control (*P*<0.001). Interestingly, all diabetic groups receiving either metformin or extract demonstrated a remarkable reduction of blood glucose versus the STZ group (*P*<0.001, for all groups; Figure 1). STZ-Mix extracts demonstrated no significant difference with STZ-metformin groups, whereas there was significant difference with the STZ-group in blood glucose level and even better than groups receiving the extracts alone (*P*<0.001). 

***Mix of extract improved spatial learning and memory impairment in diabetic rats***

**Figure 2:**
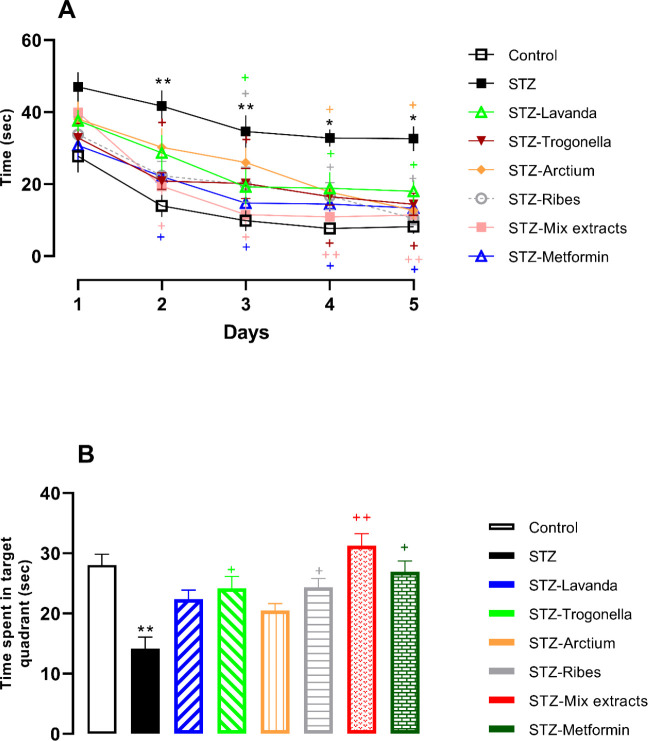
A) latency to find the platform during 5 days and B) time spent in target quadrant in probe day

Based on the statistical analysis, times spent to find the hidden platform in the STZ group in both trial and probe tests were significantly different from controls (*P*<0.05 and *P*<0.01, respectively; Figure 2), while groups receiving metformin and extracts or mixed-extracts demonstrated shorter time spent versus the STZ group (*P*<0.05), and also in the probe day metformin and extracts significantly had increased time spent in the target quadrant (*P*<0.05). Surprisingly, on the probe day, STZ-Mixed extracts demonstrated better results than extracts administrated alone and spent more time in the target quadrant in comparison with the STZ group (*P*<0.01). 

***Mix of extract improved non-spatial learning and memory impairment in diabetic rats***

**Figure 3:**
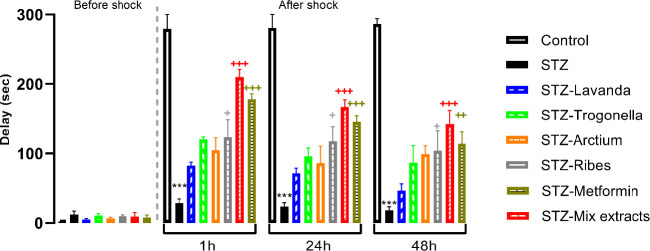
Comparison of latency to enter the dark compartment in different experimental groups 1, 24 and 48 hrs after shock

Results have shown a significant difference between groups receiving STZ and control for 1, 24, and 48 hr post-shock (*P*<0.001, *P*<0.001, and *P*<0.01 respectively). In fact, STZ groups demonstrated significant time latency to enter the dark chamber 1, 24, and 48 hr post-shock versus control, whereas groups receiving metformin had significantly decreased the number of entrances and increased time latency into the dark chamber compared to the STZ group (*P*<0.001 and *P*<0.01 for 24 and 48 hr post-shock, respectively). Interestingly, all groups receiving extracts demonstrated a significant reduction to enter the dark chamber in comparison with the STZ group (*P*<0.05 for all), while in groups receiving mixed extract, the negative effect of STZ was ameliorated better than administration of extract alone (*P*<0.001, *P*<0.01, and *P*<0.001 for 1, 24, and 48 hr post-shock, respectively; Figure 3). 

***Mix of extracts improved hippocampal oxidative/anti-oxidative imbalance in diabetic rats***

**Figure 4:**
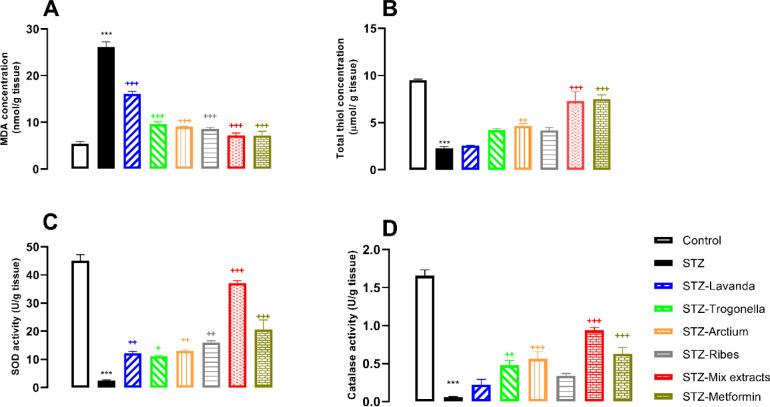
Comparative analysis of Glial fibrillary acidic protein (GFAP) (A), amyloid-B (B), and Brain-derived neurotrophic factor (BDNF) (C) parameters in hippocampal tissues between groups

MDA concentration in the hippocampus tissue of groups receiving STZ was significantly higher than in controls (*P*<0.001; Figure 4A), while groups receiving either metformin or extracts and their mixture were able to decrease MDA as a known oxidative stress biomarker in comparison with the STZ-group (*P*<0.001, for all; Figure 4A). On the contrary, total thiol content in the group receiving STZ demonstrated a significant reduction versus control (*P*<0.001; Figure 4B), while metformin and other groups receiving extract or their mixture demonstrated significant increase in total thiol against the STZ group (*P*<0.001, for all; Figure 4B). 

SOD as an anti-oxidative biomarker demonstrated significantly higher activity in groups receiving metformin and extract or their combination against the STZ group (*P*<0.001 for all), while the STZ group demonstrated lower activity in comparison with control (*P*<0.001; Figure 4C). Catalase activity remarkably decreased in the STZ group, whereas metformin, Arctium, and mixed extracts significantly increased catalase activity versus the STZ group (*P*<0.001, *P*<0.01, and *P*<0.001, respectively; Figure 4D).

***Mix of extracts improved cortical oxidative/anti-oxidative imbalance in diabetic rats***

**Figure 5:**
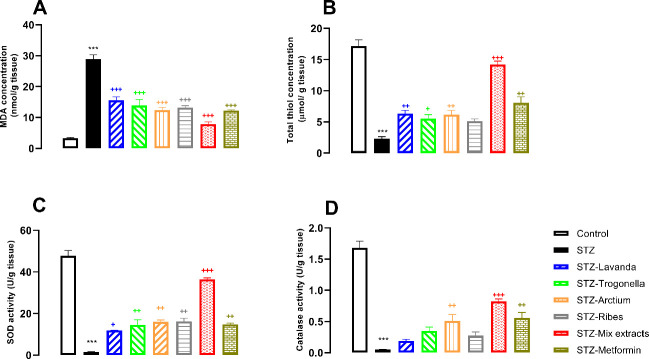
A) MDA, B) total thiol content, C) SOD activity and D) catalase activity, comparative analysis between groups in cortical tissues

According to statistical analysis, the concentration of MDA in the STZ group was significantly higher than in controls (*P*<0.001), whereas the groups receiving extracts, mixed-extract, or metformin had remarkably decreased MDA levels in comparison with the STZ group (*P*<0.001; Figure 5A). On the contrary, total thiol content in the STZ group notably decreased versus controls (*P*<0.001; Figure 5B), while treatment of diabetic rats with either metformin or mixed extracts significantly increased total thiol against the STZ group (*P*<0.001 for both). Also, the groups receiving Lavanda, Arctium, or Trogonella extracts had increased total thiol content (*P*<0.01, *P*<0.01, and *P*<0.05), however, the group receiving Ribes extract demonstrated insignificant difference versus the STZ group. 

Significant reduction of SOD activity was observed between the control and the STZ group (*P*<0.001; Figure 5C), whereas the groups receiving either metformin, extracts, or mixed extracts significantly increased SOD activity in comparison with the STZ group (*P*<0.01 for all except Lavanda with *P*<0.05; Figure 5C). According to the present findings, catalase activity in the STZ group significantly decreased versus control (*P*<0.001; Figure 5D), while groups receiving metformin or mixed-extracts demonstrated a notable increase in enzyme activity as compared to the STZ group (*P*<0.001 and *P*<0.01; Figure 5D, respectively). The group receiving Arctium extract demonstrated significant differences in comparison with the STZ group (*P*<0.01; Figure 5D), while the rest of the groups receiving other extracts revealed insignificant differences with the STZ group.

***STZ reduced BDNF and increased GFAP and amyloid-B concentrations in hippocampal tissues: Improvement by mixed extracts treatment***

**Figure 6:**
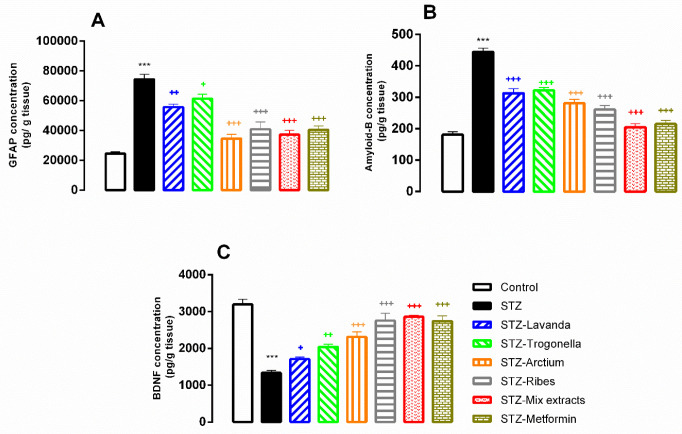
Comparative analysis of GFAP (A), amyloid-B (B), and BDNF (C) parameters in hippocampal tissues

Further assessments in hippocampal tissue samples revealed that STZ reduces the BDNF concentration as compared to control animals (*P*<0.001; Figure 6A). In contrast, GFAP and amyloid-B levels were significantly elevated in the same tissues taken from the STZ-treated subjects (*P*<0.001; Figure 6B and C). It should be noted that in the case of BDNF, all treatments could prevent the STZ effects (*P*<0.05, *P*<0.01, and *P*<0.001; Fig6A), also, on amyloid-B and GFAP, all treatments were found to be effective (*P*<0.05, *P*<0.01, and *P*<0.001; Figure 6B and C).

## Discussion

Regarding a variety of complications associated with microvascular and macrovascular diseases induced by diabetes, in addition to implementation of prevention programs, medicinal plants as a future source of new drugs should be considered (37). So, considering the rich source of herbal medicines in Iran, in the present study, we showed the positive impact of different well-known extracts including *Lavandula*, *Arctium*, *Trigonella*, *Ribes*, and their combination on biochemical biomarkers such as blood glucose levels, oxidative stress over anti-oxidant mediators, and learning-memory behavioral analysis in STZ -induced diabetic models in rats. The results of MWM indicated that the time spent to find the platform was significantly increased as compared to the control group and the time spent in the target quadrant notably decreased in the probe test. Moreover, numerous available literature demonstrated learning and memory impairment in STZ-induced diabetes rats (38-40). In the next step, the PA test was done and consistently the results supported the exacerbation of learning and memory impairment in diabetic subjects. Technically, this was revealed by the reduction of the animal’s delay to enter the dark compartment aftershock (39). Up to this step, our results were in line with previous studies suggesting that STZ causes learning and memory impairment in rodents (41).

However, regarding some evidence supporting the beneficial effects of *Lavandula*,* Arctium, Trigonella*,* and Ribes *on diabetes (23, 28, 42-44), the present study aimed to investigate whether the negative effects of diabetes induced by STZ, especially on learning and memory could be prevented by either these extracts when administered alone or in mixture with lower doses. In fact, to the best of the author’s knowledge, there is no scientific study that has been conducted to evaluate their synergistic effects on learning and memory impairment induced by STZ. For this purpose, extracts and their mixture were administered to diabetic rats, and behavioral assessments of learning and memory were carried out. Interestingly, results showed that extract therapy ameliorates diabetes-induced changes in behavioral indices measured in MWM and PA (Figures 2 and 3), and more importantly, the mixture of extracts can reduce the effects of diabetes on memory as well as metformin. In this regard, it should be noted that almost in all experiments, the effects of these extracts were found to be more potent at higher doses, which is in agreement with previous findings (45, 46). 

As would be expected, in all biochemical and behavioral test analyses, extracts and their combination in comparison with diabetic rats demonstrated significant reduction of blood glucose and oxidative stress mediators like MDA, while anti-oxidant mediators including total thiol content and catalase/SOD activity notably increased. It is noteworthy that the combination of the extracts in lower doses was more effective than the extract administrated alone which could be related to the synergistic effect of compounds available in the extracts, which means these herbal plants and their active chemical constituents can be used for the management of diabetic patients. Numerous studies demonstrated the anti-diabetic effect of Lavandula related to its anti-oxidant, anti-inflammatory, or anti-hyperglycemic activity through inhibition of α-glycosidase enzyme which is consistent with the present results (47). Also, some animal studies provided evidence supporting the positive effect of Lavandula on learning-memory impairment induced by diabetes (48). Furthermore, there are numerous studies demonstrating a wide range of pharmacological effects of Arctium including anti-viral, hypolipidemic, anti-inflammatory, and anti-diabetic related to fructooligosaccharide as an effective constituent of Arctium, which has gained considerable attention against DM. Consistent with the present study, Trigonella as well as Ribes extracts have also shown positive effects on the reduction of blood glucose, and improvement of learning memory in diabetic rats (42, 49). 

Therefore, according to the results, the possible therapeutic effects of *Lavandula*, *Arctium*, *Trigonella*, *Ribes*, and their combination were almost confirmed and it was found that the herbal medicine used in the present study can prevent STZ-induced oxidative stress by suppression of oxidative parameters and enhanced antioxidant parameters (Figures 4 and 5). Our findings are in line with other studies that showed these extracts alone can improve oxidative/anti-oxidative imbalance in different species and human body organs (50-53). Furthermore, the present findings demonstrated that the mixture of these extracts with a quarter dose can improve the harmful effects of diabetes as much as metformin. Interestingly, high doses of the extracts were effective in most of the parameters, but they could not be effective in all parameters, while a quarter doses of the extracts in the form of a mixture can be effective in all parameters, which can be explained by synergistic effects of the extracts; however, it needs further investigation to decide more confidently.

Moreover, other parameters including amyloid-B, BDNF, and GFAP which have a promising role in neurodegenerative disorders were significantly changed. In this respect, numerous studies have shown that diabetes can cause memory disorders by increasing the amount of amyloid-B. Recently, it has been reported that systemic STZ injections increase amyloid genesis by activating beta and gamma-secretase, resulting in an increase in the level of amyloid-B-1-42 in the hippocampus (54, 55). We showed that these extracts and their mix can decrease elevated levels of amyloid-B in diabetic rats. Some of these extracts have decreasing effects on amyloid-B. It was shown that Lavandula can decrease the level of amyloid-B (56). 

 Therefore, based on these findings and the results of the present study, elevated levels of GFAP can be considered a possible mechanism for memory impairment caused by STZ. We have shown for the first time that STZ-induced diabetes can increase the level of GFAP. In addition, our results showed that the studied extracts and their mixture can prevent the increase of this parameter.

BDNF is a molecule that is abundantly present in the hippocampus and it has been shown that the amount of this substance is reduced in diabetes and can be effective in the process of memory impairment caused by diabetes (57). We also showed that diabetes induced by STZ can reduce the amount of BDNF. Among the extracts that we used in this study, only two, *Lavandula *and *Arctium *extracts, have been shown to have a positive effect on BDNF levels (58, 59).

The most important point of the current study was the use of extract combination with minimum dose. Considering that there are numerous factors including lack of insulin, hyperglycemia, overproduction of pro-inflammatory cytokine and oxidative stress mediators along with reduction of anti-oxidant or anti-inflammatory reagents play pivotal roles in the development and progression of diabetes, these well-known extracts and especially their mixture can be explored to raise new hope in the treatment of this complicated disease.

Data presented as mean±standard error of the mean (SEM). ***P<0.001 vs control group, ^+++^ *P<*0.01 vs streptozocin (STZ) group

Data presented as mean±standard error of the mean (SEM). n=10. *P<0.05 and **P<0.01 vs control group, ^+^*P<*0.05, and ^++^*P<*0.01 vs streptozocin (STZ) group

Data presented as mean±standard error of the mean (SEM). n=10. ***P<0.001 vs control group, ^+^*P<*0.05, ^++^*P<*0.01, and ^+++^*P<*0.001 vs streptozocin (STZ) group

Data presented as mean±standard error of the mean (SEM). n=10. ***P<0.001 vs control group, ^+^*P<*0.05, ^++^*P<*0.01, and ^+++^*P<*0.001 vs STZ group

MDA: malondialdehyde, SOD: superoxide dismutase, STZ: streptozocin

Data presented as mean±standard error of the mean (SEM). n=10. ***P<0.001 vs control group, +*P<*0.05, ^++^*P<*0.01, and ^+++^*P<*0.001 vs STZ group

Eth: ethanol, MDA: malondialdehyde, SOD: superoxide dismutase, STZ: streptozocin

Data presented as mean±standard error of the mean (SEM). n = 10. ***P<0.001 vs control group, +*P<*0.05, ^++^*P<*0.01, and ^+++^*P<*0.001 vs STZ group

Eth: ethanol, MDA: malondialdehyde, SOD: superoxide dismutase, STZ: streptozocin

## Conclusion

Considering that a wide variety of complications in many key organs induced by diabetes leads to not only physical and mental problems, but also imposes a very high economic burden on both individuals and society, so, higher efforts to find new anti-diabetic medicines with higher efficacy and lower side effects is necessary. This study introduced some novel herbal medicines as effective in diabetes, and it is noteworthy that the mixture of extracts with minimum dose was more effective than extracts administrated alone.

## Authors’ Contributions

F B designed and performed experiments, analyzed data, and co-wrote the paper. J F designed experiments, analyzed data, and co-wrote the paper. S K interpreted the data and co-wrote the paper. MS HA performed experiments and co-wrote the paper. MH E performed analyses.

## Data availability statement

The data that support the findings of this study are available from the corresponding author, upon reasonable request.

## Conflicts of Interest

We declare that we have no conflicts of interest.
